# You don’t have the guts: a diverse set of fungi survive passage through *Macrotermes bellicosus* termite guts

**DOI:** 10.1186/s12862-020-01727-z

**Published:** 2020-12-09

**Authors:** Nick Bos, Leandro Guimaraes, Romen Palenzuela, Justinn Renelies-Hamilton, Lorrie Maccario, Simon Kolotchèlèma Silue, N.’golo Abdoulaye Koné, Michael Poulsen

**Affiliations:** 1 Department of Biology, Section for Ecology and Evolution, University of Copenhagen, Universitetsparken 15, Building 3, 1st floor, 2100 Copenhagen East, Copenhagen, Denmark; 2 Department of Biology, Section of Microbiology, University of Copenhagen, Universitetsparken 15, Building 1, 1st floor, 2100 Copenhagen East, Copenhagen, Denmark; 3 Unité de Recherche en Ecologie Et Biodiversité (UREB), Université Nangui Abrogoua, UFR Des Sciences de La Nature (UFR-SN), 28 BP 847 28, Abidjan, Côte d’Ivoire; 4 Centre de Recherche en Écologie (CRE), Station de Recherche en Ecologie du Parc National de La Comoé, Bouna, Côte d’Ivoire

**Keywords:** Antimicrobial defence, Macrotermitinae, Mycobiome, *Pseudoxylaria*, *Termitomyces*

## Abstract

**Background:**

Monoculture farming poses significant disease challenges, but fungus-farming termites are able to successfully keep their monoculture crop free from contamination by other fungi. It has been hypothesised that obligate gut passage of all plant substrate used to manure the fungal symbiont is key to accomplish this. Here we refute this hypothesis in the fungus-farming termite species *Macrotermes bellicosus*.

**Results:**

We first used ITS amplicon sequencing to show that plant substrate foraged on by termite workers harbour diverse fungal communities, which potentially could challenge the farming symbiosis. Subsequently, we cultivated fungi from dissected sections of termite guts to show that fungal diversity does not decrease during gut passage. Therefore, we investigated if healthy combs harboured these undesirable fungal genera, and whether the presence of workers affected fungal diversity within combs. Removal of workers led to a surge in fungal diversity in combs, implying that termite defences must be responsible for the near-complete absence of other fungi in functioning termite gardens.

**Conclusions:**

The rapid proliferation of some of these fungi when colonies are compromised indicates that some antagonists successfully employ a sit-and-wait strategy that allows them to remain dormant until conditions are favourable. Although this strategy requires potentially many years of waiting, it prevents these fungi from engaging in an evolutionary arms race with the termite host, which employs a series of complementary behavioural and chemical defences that may prove insurmountable.

## Background

Millions of years prior to human agriculture, monoculture fungus farming evolved in attine ants (tribe: Attini) and fungus-growing termites (subfamily: Macrotermitinae) in South America and Africa, respectively. Monoculture farming predictably increases risks of epidemic infections [1, 2], while high genetic diversity buffers resistance against disease [3]. This ‘monoculture effect’ is well-documented in human agriculture [4] and in leaf-cutting ant monocultures, where specialized and potentially virulent fungal pathogens tend to invade the fungal cultivar [5–7]. However, in sharp contrast to humans and ants, fungus-farming termites appear to have successfully overcome the problem of monocultures being susceptible to disease invasion [8].

Fungus farming in termites originated about 31 million years ago in the termite subfamily Macrotermitinae (Termitidae) [9]. The termites cultivate a fungal symbiont crop in the genus *Termitomyces* (Basidiomycota: Lyophyllaceae) on foraged plant biomass. The obligate symbiosis has diversified to 11 termite genera cultivating more than 40 described *Termitomyces* species [10, 11]. The fungus is provided with shelter, optimal growth conditions [12] and a supply of pre-digested plant substrates [13] foraged on by termite workers. In return, it provides termite nourishment from plant sources, which the termites cannot digest on their own [14]. The manuring of fungus combs is through a strict process, where older workers forage on decaying plant biomass outside the nest, which is passed on to younger workers within the nest, who ingest it along with asexual *Termitomyces* spores in a ‘first gut passage’ [15, 16]. This gut passage is rapid and serves to efficiently mix *Termitomyces* spores with the macerated substrate, and this mix is deposited as fresh fungus comb [15], on which *Termitomyces* grows to decompose the plant substrate, produce mycelium and new asexual spores.

The maintenance of a dense *Termitomyces* monoculture symbiont population creates an environment within which diseases are predicted to be able to rapidly spread [17, 18]. However, despite being in close contact with substrates containing potentially competing fungi [19], intact fungus-growing termite colonies do not appear to have antagonistic fungi growing within fungal combs, with 99.9% of Internal Transcribed Spacer (ITS) amplicon reads being of the mutualistic *Termitomyces* [8]. However, if colonies are physically compromised or workers are removed from the fungus comb, *Termitomyces* is rapidly infested and overgrown by generalist fungi [20] and members of the specialist fungal sub-genus *Pseudoxylaria* (Ascomycota: Xylariaceae) [21–27].

A number of complementary defence mechanisms appear to contribute to keep fungus gardens free from other fungi [8, 28]. These include termites avoiding antagonists [29], burying unwanted fungi [30], and utilising antimicrobial chemical compounds of termite [22], *Termitomyces* [8, 31, 32] and bacterial [8, 20, 33, 34] origins. In addition, it has been proposed that the obligate first gut passage of all plant substrate used to manure the fungus could serve as a filtering—potentially fungicidal step—accounting for the very low prevalence of non-*Termitomyces* fungi within fungus combs [16, 35].

Here we use culture-independent amplicon sequencing to characterize the fungal communities present within substrate foraged on by workers to examine which fungal genera the symbiosis is exposed to during foraging. Secondly, we compare the diversity of culturable fungi that can be obtained from termite foreguts, middle section (midgut, paunch and colon) and rectum. We hypothesized that foraging material contains a high diversity of fungi and that fungal diversity would decrease during gut passage. As we did not find a reduction in fungal diversity during gut passage, we subsequently used amplicon sequencing to investigate if healthy combs with workers present harboured the fungal genera that we obtained from guts, and if the removal of workers would lead to a surge in their growth.

## Results

### Assessment of fungal diversity in foraging substrates

**Fig. 1:**
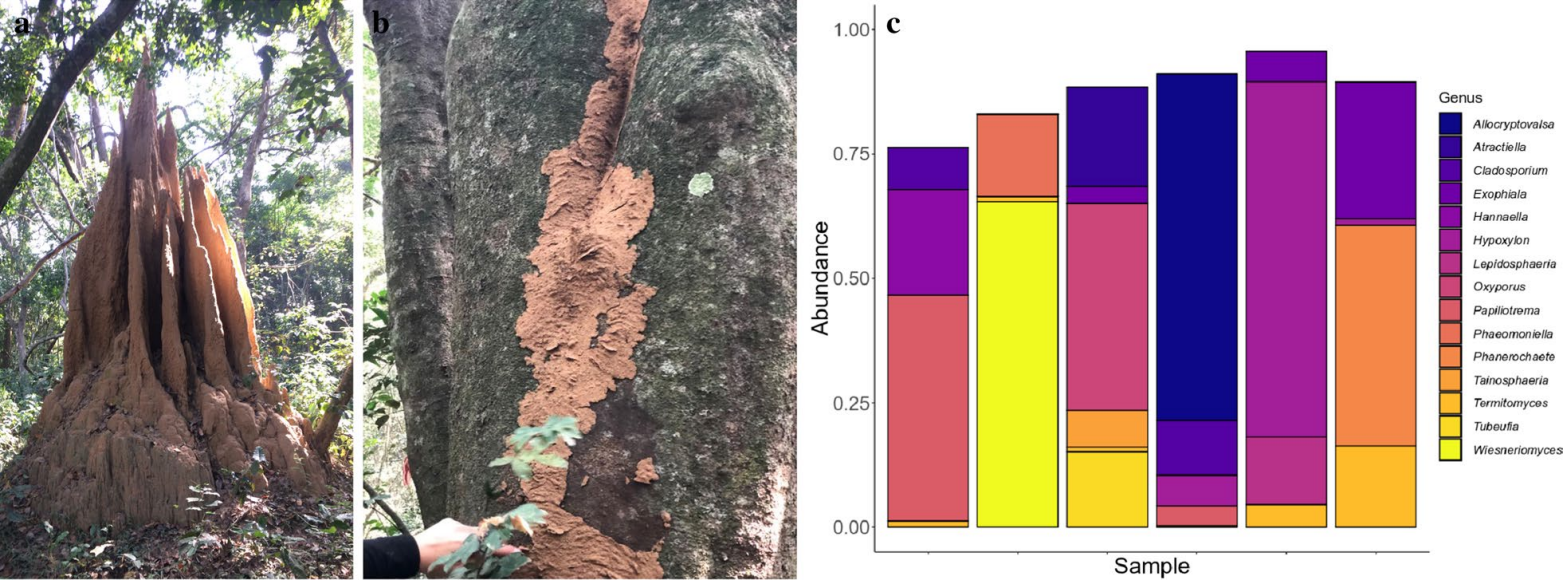
**a** A *Macrotermes bellicosus* colony in Côte d’Ivoire (Photo: MP), **b** A foraging sheet constructed by workers covering the substrate (Photo: RP), **c** Relative abundance of the 15 most common fungal genera in the foraging substrates

We obtained 7,758,822 (mean ± SE: 83,428 ± 5,796) clean fungal ITS reads from all sequenced samples (experiment a + experiment c, Additional file 1: Table S2). Of eight foraging substrates that identified to *M. bellicosus*, six were successfully sequenced, resulting in 271,227 (mean ± SE: 45,204 ± 12,813) fungal reads. We restrict our main text presentation of results to *M. bellicosus*, but show results for all species in Additional file 1: Table S2 and Additional file 2: Figure S2. 1,403,519 (mean ± SE: 50,125 ± 6,828) clean reads were used to assess worker impact on fungus comb health (experiment c). Sequences from both experiments were assigned to 1,970 unique Amplicon Sequence Variants (ASVs), of which 96.4% were identified to phylum, 94.9% to class, 93.0% to order, 89.5% to family and 86.2% to genus level. Foraging substrates were highly variable in their composition of fungal genera, with no clear indications of dominant genera (Fig. 1c).

### Diversity of culturable fungi in three segments of the gut

**Fig. 2:**
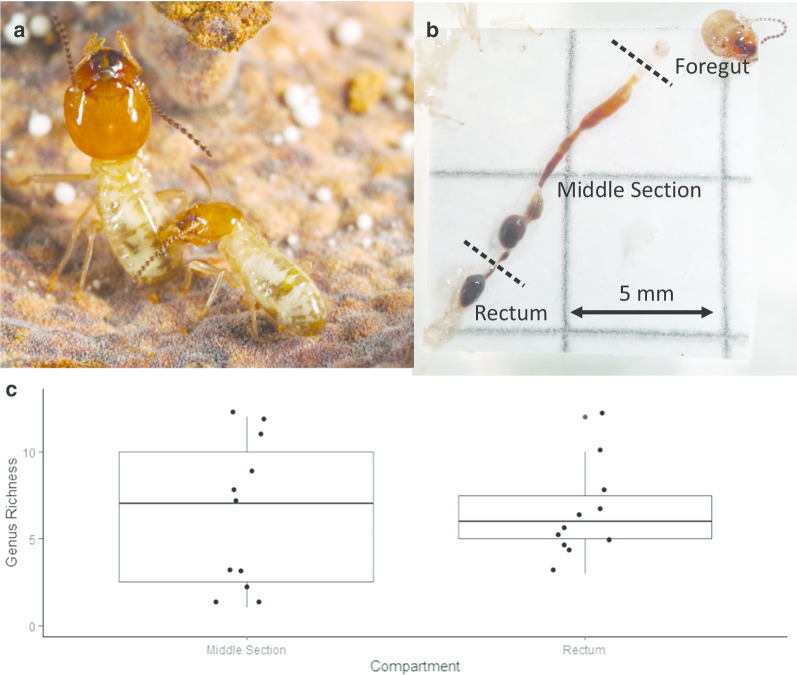
**a** Minor worker grooming a major worker (Photo: NB). **b** Dissected gut showing the division into foregut, middle section consisting of midgut, paunch and colon, and rectum (Photo: LG). **c** Mean ± SD of observed fungal genus richness per gut compartment. Each dot represents one sample

Sequencing our isolates revealed 51 fungal genera, from which 10 were found in foreguts, 30 in the middle section, and 33 in the rectum (Additional file 3: Table S3). During dissections, the foregut was often empty (Fig. 2b), and did not provide a satisfactory amount of material, as the genus richness was ~ 3 times less than in the following compartments. Therefore, we based subsequent analyses on the middle section and rectum only. The separation of half of the samples into smaller sub-samples of five and ten guts did not affect the number of genera isolated (LM, separation, F = 0.3315, p = 0.5739). Furthermore, there was no significant difference in genus richness between the middle section and rectum (GLM, compartment, F = 0.0361, P = 0.8520, Fig. 2c). There was no effect of nest of origin on genus richness (GLM, nest, F = 0.5298, p = 0.7503), nor on species composition (PERMANOVA, 999 permutations, R^2^ = 0.2711, F = 1.190, p = 0.21).

### Effect of worker population size on fungus comb health

**Fig. 3:**
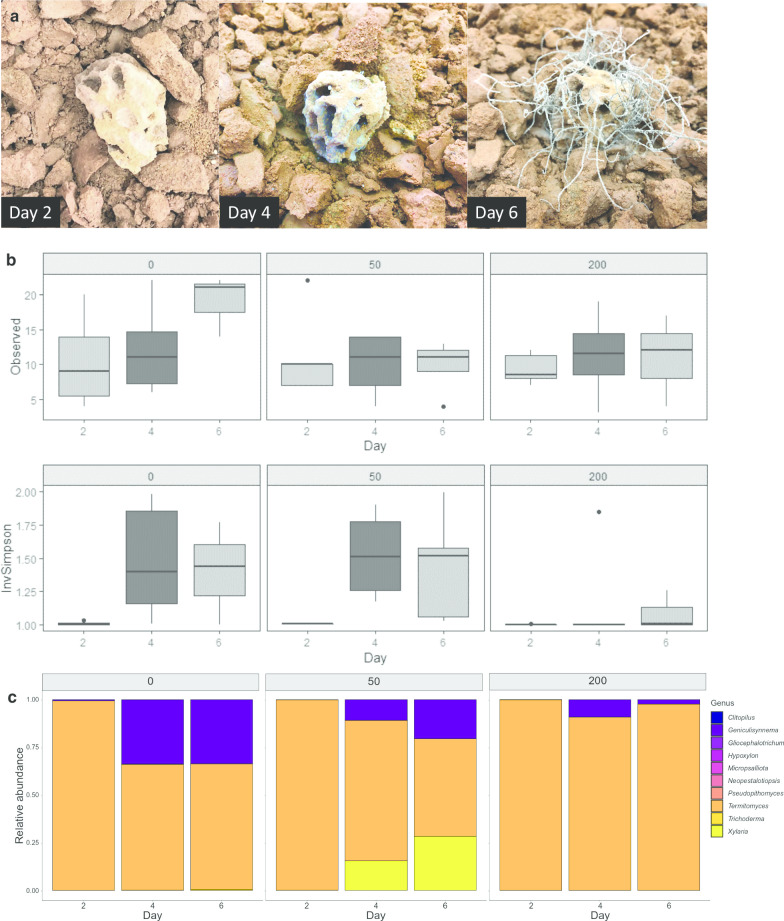
**a** An example of fungal growth during the three sampling points, with clear presence of non-*Termitomyces* growth on day four and complete *Pseudoxylaria* overgrowth on day 6. Images taken from colony IC0031 with 50 workers. Photos: RP. **b** Box plots of fungal genus diversity, calculated as the inverse Simpson index (top) and observed richness (bottom) as a function of the number of workers present (0, 50 or 200) and time (2, 4 and 6 days). **c** Relative abundance of the 10 most common genera present in the fungus comb. Although the comb mostly consists of Termitomyces, the genera Xylaria and Geniculisynnema (which has been proposed to be reclassified to Xylaria [36]) quickly rise in dominance when workers are absent

We investigated the effect of worker number on fungal diversity in fungus combs. Forty-two of our comb samples were successfully sequenced, allowing for comparison of fungal diversity across sub-colonies with 0, 50 or 200 workers. Fungal diversity, as measured by the Inverse Simpson index, increased over time (GLM, Day, LR χ^2^ = 18.70, p < 0.0001, Fig. 3b), irrespective of worker numbers; however, the presence of workers significantly reduced this increase in fungal diversity (GLM, workers, LR χ^2^ = 7.303, p = 0.0069, Fig. 3b). In contrast, observed species richness was neither affected by day (GLM, Day, LR χ^2^ = 1.027, p = 0.5986, Fig. 3b) or the number of workers (GLM, Day, LR χ^2^ = 0.8189, p = 0.3655, Fig. 3b). 79% of the fungal genera found in the foraging material were also identified in fungus combs. On day 6, fungus combs were dominated by *Xylaria* and *Geniculisynnema*, which has been proposed to be reclassified as *Xylaria* [36].

## Discussion

Maintaining a monoculture crop fungus for years on decaying plant biomass without succumbing to pathogen outbreaks is a challenge that fungus-growing termites appear to successfully achieve. This must require very effective defence mechanisms, and it was proposed already almost a decade ago that obligate gut passage of all plant substrate could be an effective filter for removing environmental competitors or antagonistic fungi of *Termitomyces* [16]. Our findings strongly suggest that this is not the case: substrates harbour a high diversity of fungi, and we find no evidence that fungal diversity decreases as we move from the middle to the latter section of the gut. As expected, we did however confirm previous findings that fungus combs are dominated by *Termitomyces* [8], and that the presence of workers is vital for ensuring that combs remain free from other fungi [28]. We discuss our results in the context that environmental fungi enter termite fungus combs, where alternative mechanisms prevent their proliferation until the integrity of the environment and worker presence for one reason or another is compromised.

Foraging *M. bellicosus* workers encase their foraging material in a thin sheet of soil and close it off from the environment (Fig. 1b), but the fungal diversity we observed below these sheets was appreciable, suggesting that this environment is not sanitised by the foraging workers. Unsurprisingly, many of the most abundant genera, such as *Hypoxylon*, *Allocryptovalsa* and *Wiesneriomyces* (Fig. 1c), are wood decomposers [37–39] and they may thus compete with *Termitomyces* if they enter fungal combs. Although in low abundance, members of the family Xylariaceae appeared in all *M. bellicosus* foraging materials. BLASTn search indicated that 31% of these Xylariaceae amplicons most closely resembled members of the termite-specific sub-genus *Pseudoxylaria* [25], while 69% matched better with free-living *Hypoxylon*. All samples contained ASVs that most closely resembled *Pseudoxylaria,* ranging from 1 to 4 (average = 2.2) different ASVs per foraging site. It is still unknown how *Pseudoxylaria* spreads from colony to colony, but this raises the possibility that spreading could take place through sharing of foraging tunnels, which is conceivable as larger termite mounds often host multiple farming termite species and even genera ([40, 41]; personal observations). *Termitomyces* was also present in some foraging sites (Additional file 1: Table S2, Additional file 2: Figure S2). While this could be due to accidental deposition by the foraging termites, we cannot rule out it is due to contamination, as *Termitomyces* was present in relatively high abundance and grouped to identical ASVs in many samples, even after filtering. Irrespective of whether or not *Termitomyces* is present in foraging sheets, it is clear that these foraging substrates are populated with a diversity of fungi, including potential competitors or antagonists of *Termitomyces*.

Contrary to our expectations, fungi ingested by the termites were not eliminated during gut passage. Although we limited our search to culturable fungi, we found no evidence of any fungicidal effects of gut passage, suggesting that foraging material deposited on the fungus comb as faeces is not sterile, and that gut passage most likely primarily serves to mix substrate with asexual *Termitomyces* spores [15, 16]. Although 99.9% of amplicon reads of our subsequent fungus comb analyses were *Termitomyces* (Additional file 2: Figure S3), 79% of the fungal genera that we identified from foraging materials were identified in low relative abundances within fungus combs. The 16 fungal genera unique to the fungus comb were all associated with soil and plant material, and thus likely picked up during termite foraging. Still, the 79% overlap is remarkable given the relatively low number of sites and colonies used, and even more so as foraging sites were rarely to our knowledge used by the colonies included in the sub-colony experiment. This speaks to the consistent presence of these fungi in the termite environments. Their minute abundance within fungus combs confirms previous work that the fungus combs are consistently and vastly dominated by *Termitomyces* [8], so that mechanisms must be in place that preclude proliferation of potential competitors brought in when termites forage.

The fungus combs that forage-dwelling fungi enter is a complex environment that plays at least some role in defence, as growth of other fungi is delayed for at least two days in the absence of workers (Fig. 3b). The mutualistic fungus *Termitomyces* produces compounds with antifungal properties [8, 31, 32], which together with compounds of termite or bacterial origins [8, 33, 42] forms complex comb chemical mixtures. Although these complex communities of chemical compounds appear to suppress some putative antagonists, their functions and targets remain almost unequivocally unknown. However, antifungal compounds are unlikely to provide the full explanation for effective avoidance of non-*Termitomyces* fungal growth. The maintenance of an abiotic environment that is optimised for *Termitomyces* growth, coupled with a mode of substrate inoculation that ensures dense *Termitomyces* spore presence at the earliest stages of plant biomass decomposition, likely provide the mutualistic fungus a key competitive edge. This is analogous to the frequency-dependent selection that has been shown in the lab to help ensure that individual colonies maintain a single *Termitomyces* monoculture [1], and this could assist in ensuring effective dominance of the fungal crop within colonies in the wild. However, although this provides a competitive edge over other fungi, it is not enough to stop fungi from overgrowing the fungus comb when the termite worker force is reduced or absent, as our sub-colony experiment confirmed that worker presence is required to maintain comb homeostasis. Although the mechanisms are not fully understood, the causes are likely multiple. Oral secretions have been found to be fungistatic in several termite species [22, 43], and workers might use these to sanitise freshly-deposited faecal matter. Indeed, grooming of freshly deposited faecal pellets has been observed in *Odontotermes* sp. (Hongjie Li, personal communication), and faecal pellets express fungistatic activity in a lower termite [44]. Furthermore, as workers are in continuous contact with the fungus comb, they likely passively deposit cuticular lipids [45], which harbour antimicrobial functions in many insects, including in lower termites [46, 47]. Lastly, *Odontotermes obesus* workers have been found to bury patches of infected fungus comb [30], which could act as a final defence once antagonists start growing.

In the presence of workers, combs are dominated by *Termitomyces*, but when colonies are compromised by their removal, competitors and mycopathogens quickly overgrow combs (Fig. 3). Although many foreign species of fungi enter the fungus comb, only relatively few of these will dominate the fungus comb in the absence of workers, suggesting that many saprophytic fungi are not adapted to compete with *Termitomyces* within nests. The ones that do take over the fungus comb in the absence of workers are detected in low numbers in healthy fungus comb, and are thus likely suppressed but not eradicated from combs. This mirrors adaptations in members of the sub-genus *Pseudoxylaria* [26], which are specialists on termite fungus combs, but that do not grow or display antagonism in thriving colonies, yet will quickly overtake a colony once it has been compromised. This hypothesised sit and wait strategy has been proposed as a passive means with which *Pseudoxylaria* avoids defences from the termites and *Termitomyces* [26], which otherwise could eventually suppress *Pseudoxylaria* to complete removal.

## Conclusions

The question remains how and why the termite-associated *Pseudoxylaria*—and potentially other fungi—have adopted this sit and wait strategy. Although we cannot rule out effective suppression of parasites by the termites after gut-passage, our results may present an alternative hypothesis. If the main antifungal termite defence was to kill fungi during gut passage, *Pseudoxylaria* would either not be able to utilize fungus comb resources, or would have evolved resistance mechanisms in response to this defence, driven by rewarding nutrients. If the latter was true, and gut passage was the main termite defence, we would expect *Pseudoxylaria* to take over fungus combs as soon as they could bypass gut passage. However, as termites tolerate low numbers of inactive exogenous fungi, including *Pseudoxylaria*, but take actions against those that start proliferating [30], they may select for *Pseudoxylaria* strains that employ this sit and wait strategy. In this manner, coevolution between termite *Pseudoxylaria* and termites may have de-escalated virulence to prevent engaging in an evolutionary arms race: *Termitomyces* and termites tolerate *Pseudoxylaria* at low levels while the colony thrives, while it quickly consumes colony resources when chances arise. This requires that *Pseudoxylaria* remains within fungus combs in spite of the high turn-over of plant material, which would require growth to secure its foothold, but that growth is minimal to prevent detection and adverse responses from the termite and *Termitomyces* hosts.

## Methods

### Field site and study species

All collections and experiments were conducted at the Comoé National Park, Ivory Coast (8° 30′–9° 40′ N and 3° 10′–4° 20′ W). We collected *Macrotermes bellicosus*, which often creates large, spire-like nests (Fig. 1a) inhabited by a royal pair and up to one million neuters [48], consisting of major soldiers, minor soldiers, major workers and minor workers. A large part of the nest contains chimneys used for exchange of air and temperature regulation [49]. The centre of the nest consists of an ovoid area, containing the fungus combs as well as the royal chamber. Foraging is mainly done underground, and aboveground food-sources are covered by foraging sheets, under which the termites forage (Fig. 1b). The following methods section is divided into three separate experiments: (a) the assessment of fungal diversity in foraging substrates; (b) Diversity of culturable fungi in three segments of the gut; (c) Effect of worker population size on fungus comb health.

### Assessment of fungal diversity in foraging substrates

#### Collection of foraging material

Forage material was identified by having a fresh soil sheet covering a wooden substrate (Fig. 1b). We sampled the substrate from a total of 39 foraging sites by scraping off wood pieces into a sterile 50 ml falcon tube, which was transferred to a 2 ml screw-cap cryotube containing RNAlater, and stored at − 20 °C prior to DNA extraction. Workers and soldiers were collected for species identification.

#### Amplicon sequencing of fungal communities in plant substrates

Each substrate sample was homogenized in liquid nitrogen using a sterilized marble mortar and pestle. DNA was extracted using the DNeasy Plant Mini Kit (Qiagen), following the manufacturer’s protocol, and stored at − 80 °C until PCR. Amplicon sequencing libraries were prepared using a two-step PCR, targeting the ITS2 region with the degenerated primers gITS7 as the forward (modified from [50, 51]) and ITS4ngs as the reverse primer [51]. Amplification products were purified using the HighPrep PCR clean-up (MagBio Genomics, Galthersburg, USA). A second PCR reaction and PCR purification were performed to add Illumina sequencing adapters and sample-specific dual indexes (IDT Integrated DNA technologies, Coralville, USA) using PCRBIO HiFi for 15 amplification cycles and the HighPrep PCR Clean Up System for clean-up. Sample concentrations were normalized using the SequalPrep Normalization Plate (96) Kit (Thermofisher, USA), after which they were pooled and libraries were concentrated using the DNA Clean and Concentrator-5 Kit (Zymo Research, Irvine, USA). The pool concentration was determined using the Quant-iT High-Sensitivity DNA Assay Kit (Life Technologies, Carlsbad, USA) and libraries were diluted to 4 nM, the amplicon library was denatured and sequenced following manufacturer’s instructions on an Illumina MiSeq platform at the Section of Microbiology—University of Copenhagen, using Reagent Kit v3 [2 × 300 cycles] (Illumina, San Diego, USA).

#### Data analysis

Cutadapt v.2.3 [52] was used to remove primer sequences used in the first PCR (gITS7-ITS4ngs), both on the 5′ and the reverse complement on 3′ ends, and discarding read pairs for which any of the two primers could not be detected. Reads were further processed for error-correction, merging and amplicon sequence variants (ASVs) generation using the DADA2 v.1.12.1 [53] RStudio package with default parameters except trimLeft = 8, trimRight = 8, truncQ = 10 and truncLen (0,0). UNITE fungal database v.8.2 [54] was used with the assignTaxonomy function for taxonomic assignment of each ASV with default parameters. Three blanks were created before the first PCR to assess contamination. Our data revealed some overlap between ASV abundant in blanks and those present in the samples. In order to remove these contaminants, while still retaining biologically relevant sequences, relative abundances of ASVs present in these blanks were compared to relative abundances of the respective ASVs in the samples. If the ratio of the two relative abundances (ASV in sample / blank) was lower than 0.1, the ASV was marked as a contaminant and removed from the dataset [55]. Phyloseq v.1.28.0 [56] was used to handle data, calculate richness and diversity estimates and plot data, together with ggplot2 v.3.2.1 [57] in R v.3.6.2 [58].

### Diversity of culturable fungi in three segments of the gut

#### Worker collections

Six colonies of *Macrotermes bellicosus* were collected, hereafter referred to as IC0007, IC0027, IC0028, IC0030, IC0031 and IC0034. Species identification was based on mound shape and morphological characteristics of the soldiers [59]. The colonies were opened with a pickaxe and once fungus combs were visible, 25 minor workers were collected and immediately placed on ice to keep gut microbial activity to a minimum during transport back to the research station. Minor workers were chosen as they were most numerous within the nest.

#### Gut dissections

Guts were dissected on a small piece of paper, previously sterilized in 90% ethanol. The paper was then dried close to a flame, after which it was soaked in sterile saline solution (0.2% PBS in distilled water) to avoid desiccation of the guts during dissection (Fig. 2a, b). Each gut was exposed, and three compartments were separated into individual Eppendorf® tubes containing 150 µl sterile saline solution. To ensure that we could measure fungal diversity in the last step of the gut passage, we divided the gut into foregut; midgut, paunch and colon (henceforth: middle section); and rectum (Fig. 2b).

To evaluate the optimal number of guts per sample to capture the culturable fungal diversity, we divided the six nests in two groups. In the first group, each Eppendorf® contained gut segments of 25 workers, while the second group had the 25 workers distributed over three tubes with 5, 10, and 10 guts, respectively. This resulted in a total of 36 samples (12 per gut compartment). Eppendorf® tubes were placed at 4 °C, and transported to Copenhagen on ice.

#### Fungal isolations from gut segments

For the gut-passage experiment, we chose to employ a culture-dependent approach instead of community sequencing, as sequencing would still pick up on fungal spores that might have lost viability due to any fungicidal or fungistatic effects of gut passage, and would therefore be unsuitable to answer our question [60, 61]. Therefore, approximately one month after collection, the content of each Eppendorf® tube was homogenized with a sterile pestle, and diluted in distilled water containing 0.2% PBS and 1% Tween® 20, to a volume of 1000 µl and diluted to achieve 1% and 0.1% of the original concentration. The content of each resultant tube (1% and 0.1%) was divided across five Petri dishes containing Potato Dextrose Agar (PDA; 39 g/l) with antibacterials (ampicillin, 50 µl/ml, chloramphenicol 35 µl/ml and streptomycin, 100 µl/ml). Petri dishes were sealed with Parafilm and incubated at 25 °C until fungal growth was observed. Individual fungal Colony Forming Units (CFUs) were transferred to a new Petri dish (35 mm with PDA) to acquire pure cultures. In the case of many visually similar CFUs, at least three of each kind were isolated, resulting in a total of 736 fungal isolates, from which biomass was stored in glycerol (87%) / peptone (1%) at -20 °C at the University of Copenhagen. Of the 36 gut samples, eight foreguts, two middle section and one hindgut did not have any fungal growth.

#### Barcoding of fungal isolates

Fungal isolates were grouped according to macromorphology, resulting in 154 morphotypes. We extracted and sequenced at least three isolates of each morphotype per gut sample, resulting in 507 extractions. DNA of each chosen isolate was extracted using a standard Chelex protocol (Sigma-Aldrich). 20 μl PCR were prepared using 10 μl Phusion® High-Fidelity PCR Master Mix (New England Biolabs), 0.2 μl 100 × purified BSA (New England Biolabs), 0.8 μl of each primer, 6.72 μl sterile distilled water, and 2 μl template. Primers used were ITS1 and ITS4 [62]. PCR conditions were 94 °C for 3 min, 40 cycles of 94 °C for 60 s, 55 °C for 60 s and 72 °C for 90 s, followed by a final extension at 72 °C for 7 min. Following amplification, target PCR products were visualised by agarose gel electrophoresis. Purification was done by addition of 25% ExoSAP-IT™ (Applied Biosystems) to the PCR product, after which they were sequenced at Eurofins (http://www.eurofinsgenomics.eu), resulting in 270 successfully sequenced samples. Consensus sequences were generated in Geneious Prime 2020.1.2 when possible. In cases where either forward or reverse sequencing failed, only one sequence was used. The resulting sequences (n = 270) were matched to the NCBI nucleotide database using BLAST+, from which the top four hits were extracted and the best hit with a valid taxonomic genus name was kept [63]. Sequences with a query coverage lower than 70% or identities lower than 80% were discarded (n = 22), resulting in a final set of 248 genotyped isolates.

#### Statistical analyses

The results of these two dilutions were merged to capture as much diversity as possible, resulting in a list of genera found for each collected sample. The effect of grouping (25, 10 or 5 guts per sample), gut compartment and nest was tested using a general linear model. Model assumptions were checked visually and normal distribution of the residuals was tested using a Shapiro–Wilk test. To test whether nests differed in the composition of fungal genera found in the gut, we ran a permutational ANOVA (adonis function, [64]), with the fungal community as the dependent variable and nest as a fixed effect.

### Effect of worker population size on fungus comb health

#### Experimental setup

To investigate whether fungi found in the forage material are also present in the fungus comb, and to assess the role of workers on maintaining a clean fungus comb, six colonies of *Macrotermes bellicosus* were collected (IC0007, IC0027, IC0029, IC0031, IC0032 & IC0033), using the methods described in part b. Three pieces of fungus combs per colony, 10 g each, were put in separate plastic boxes (20 × 15 × 15 cm), with a layer of sterilized soil on the bottom. Each box was provided with a piece of filter paper, to which water was applied every day to ensure adequate humidity and received 0, 50 or 200 minor workers. On day 2, 4 and 6, a small piece of fungus comb was harvested from each box into a screw-cap cryotube containing RNAlater, and stored at − 30 °C until DNA extraction. DNA extraction and amplicon sequencing were done as described in part b.

#### Statistical analyses

Phyloseq v.1.28.0 [56] was used to handle data and calculate the Inverse Simpson index as well as the observed genus richness. The effect of day and the number of termites, as well as their interaction, Inverse Simpson index were analysed using a GLM with the inverse square of the index as the dependent variable, and day and number of termites as fixed effects. The effect of day and number of termites on observed richness was analysed using a similar model, but in this case the dependent variable did not need to be transformed to conform to model assumptions. Model assumptions were checked visually, and the distribution of residuals were tested using a Shapiro–Wilk normality test.

## Supplementary Information

**Additional file 1: Table S1.** Collection information of substrates and colonies used for both experiments.

**Additional file 2: Table S2.** Amplicon Sequence Variants (ASVs) identified from the MiSeq analyses of all forage substrates (experiment a, noted as ICF), as well as the fungus comb experiment (experiment c, noted as FC). The file contains four worksheets. The first is a merged file consisting of the sequence, the taxonomic assignment and the abundance of each ASV per sample. The next three worksheets are the metadata, taxonomy table and OTU table respectively, ready to be used with the Phyloseq package.

**Additional file 3: Table S3.** Fungal genera identified through isolations from guts. Each row contains a sequenced sample, its sequence, its closest match in GenBank and its metadata.

**Additional file 4: Figure S1.** ASV rarefaction curves for both amplicon sequencing sample sets. **Figure S2.** Relative abundance of the top 15 genera found in all collected foraging sites, including samples where no *Macrotermes bellicosus* foragers were found (see Additional file 4: Table S1 for details per foraging site, and Additional file 1: Table S2 for the full dataset). **Figure S3.** a) Relative abundance of the 10 most abundant genera present in fungus combs freshly collected from three colonies of *M. bellicosus*, showing vast dominance by *Termitomyces*. b) Relative abundance of the 10 most abundant non-*Termitomyces* genera present in freshly-collected combs of three colonies of *M. bellicosus* (see Additional file 1: Table S2 for the full dataset).

## Data Availability

DNA sequences: GenBank accessions MT887350—MT887596. MiSeq: SRA archive in GenBank PRJNA656413. Analyses scripts: Added as additional files.

## Abbreviations

ASV: Amplicon sequence variant
CFU: Colony forming unit
GLM: Generalized linear model
ITS: Internal transcribed spacer
LM: General linear model
LR: Likelihood ratio
PDA: Potato dextrose agar
