# Correction to: You don’t have the guts: a diverse set of fungi survive passage through *Macrotermes bellicosus* termite guts

**DOI:** 10.1186/s12862-021-01744-6

**Published:** 2021-01-25

**Authors:** Nick Bos, Leandro Guimaraes, Romen Palenzuela, Justinn Renelies-Hamilton, Lorrie Maccario, Simon Kolotchèlèma Silue, N.’golo Abdoulaye Koné, Michael Poulsen

**Affiliations:** 1 Department of Biology, Section for Ecology and Evolution, University of Copenhagen, Universitetsparken 15, Building 3, 1st floor, 2100 Copenhagen East, Copenhagen, Denmark; 2 Department of Biology, Section of Microbiology, University of Copenhagen, Universitetsparken 15, Building 1, 1st floor, 2100 Copenhagen East, Copenhagen, Denmark; 3 Unité de Recherche en Ecologie Et Biodiversité (UREB), Université Nangui Abrogoua, UFR Des Sciences de La Nature (UFR-SN), 28 BP 847 28, Abidjan, Côte d’Ivoire; 4 Centre de Recherche en Écologie (CRE), Station de Recherche en Ecologie du Parc National de La Comoé, Bouna, Côte d’Ivoire

## **Correction to: BMC Ecol Evo (2020) 20:163 **10.1186/s12862-020-01727-z

After publication of the original article [1], the authors have notified us that Figure 2 was not updated during proofs, as requested.

Originally published Fig. 2:

**Figure:**
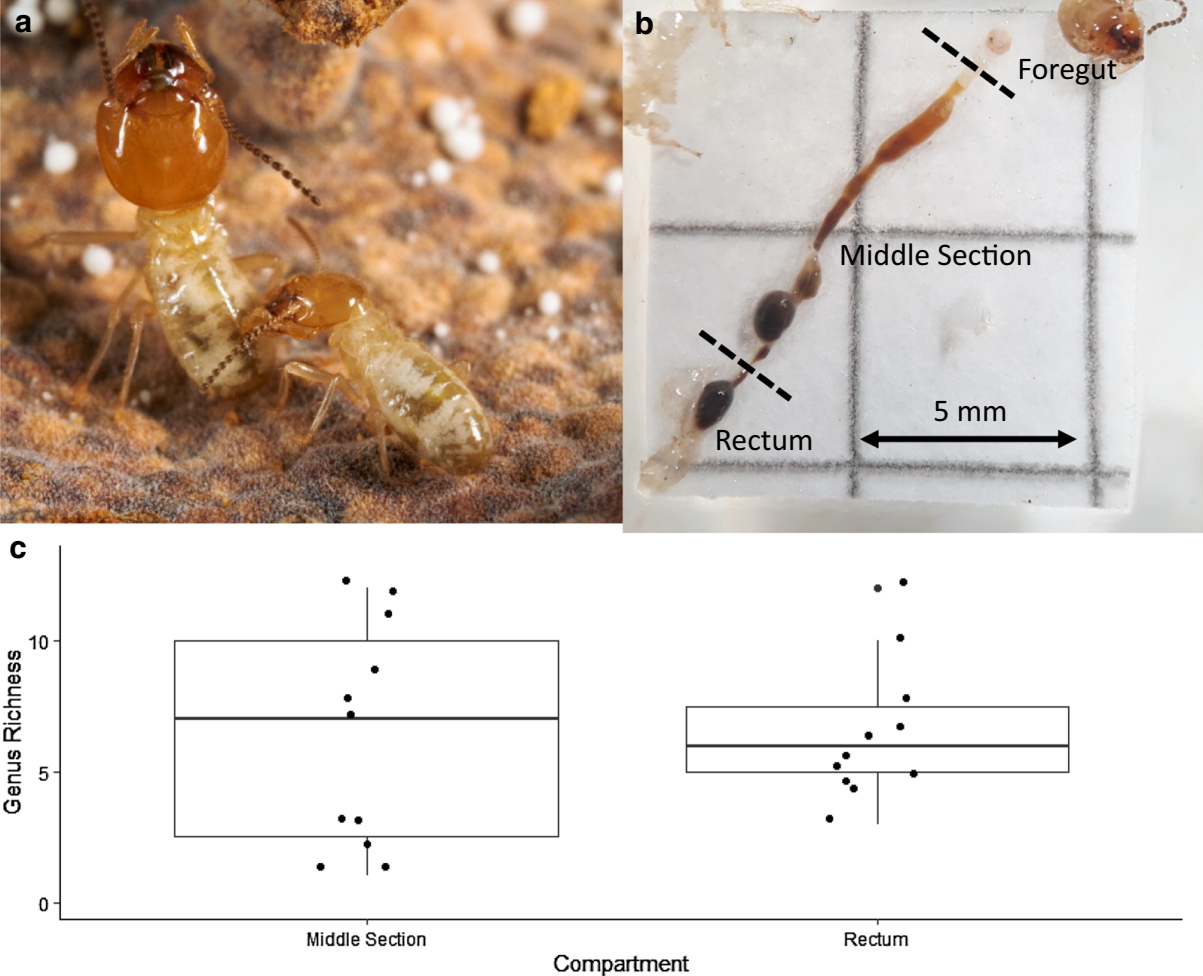

Correct Fig. 2:

**Fig. 2:**
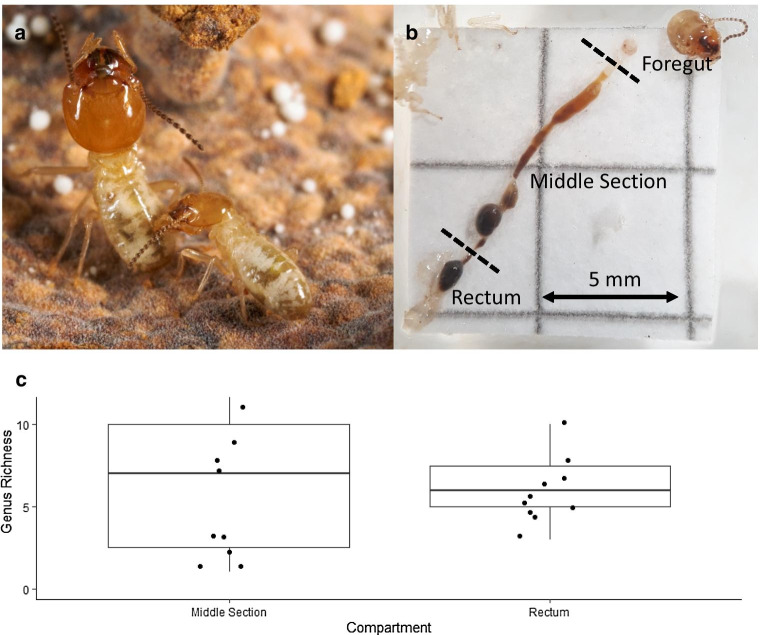
**a** Minor worker grooming a major worker (Photo: NB). **b** Dissected gut showing the division into foregut, middle section consisting of midgut, paunch and colon, and rectum (Photo: LG). **c** Mean±SD of observed fungal genus richness per gut compartment. Each dot represents one sample

The Publisher apologizes for this error and any confusion caused.

The original article has also been updated.
