# Supplementary material for: You don’t have the guts: a diverse set of fungi survive passage through Macrotermes bellicosus termite guts
Source: BMC Evol Biol. 2020 Dec 9;20:163. doi: 10.1186/s12862-020-01727-z (PMC7724875; doi:10.1186/s12862-020-01727-z)
Supplement: Supplementary file 4 — Additional file 4: Figure S1. ASV rarefaction curves for both amplicon sequencing sample sets. Figure S2. Relative abundance of the top 15 genera found in all collected foraging sites, including samples where no Macrotermes bellicosus foragers were found (see Additional file 4: Table S1 for details per foraging site, and Additional file 1: Table S2 for the full dataset). Figure S3. a) Relative abundance of the 10 most abundant genera present in fungus combs freshly collected from three colonies of M. bellicosus, showing vast dominance by Termitomyces. b) Relative abundance of the 10 most abundant non-Termitomyces genera present in freshly-collected combs of three colonies of M. bellicosus (see Additional file 1: Table S2 for the full dataset). [file 12862_2020_1727_MOESM4_ESM.docx]

**Additional file 4**

Table S1. Collection information of substrates and colonies used for both experiments.

Table S2. Amplicon Sequence Variants (ASVs) identified from the MiSeq analyses of all forage substrates (experiment a, noted as ICF), as well as the fungus comb experiment (experiment c, noted as FC). The file contains four worksheets. The first is a merged file consisting of the sequence, the taxonomic assignment and the abundance of each ASV per sample. The next three worksheets are the metadata, taxonomy table and OTU table respectively, ready to be used with the Phyloseq package.

Table S3. Fungal genera identified through isolations from guts. Each row contains a sequenced sample, its sequence, its closest match in GenBank and its metadata.

**Additional figures with legends**


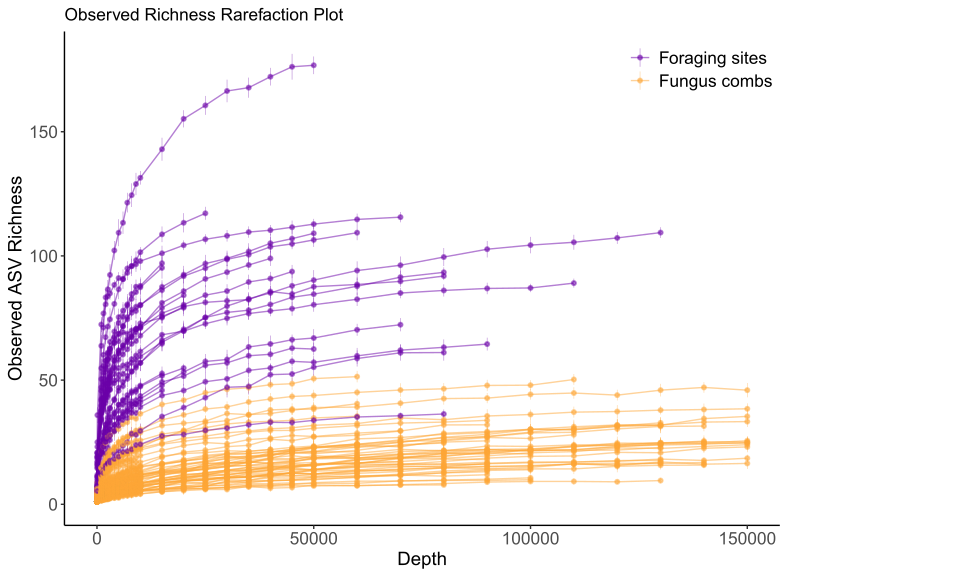


Figure S1. ASV rarefaction curves for both amplicon sequencing sample sets.


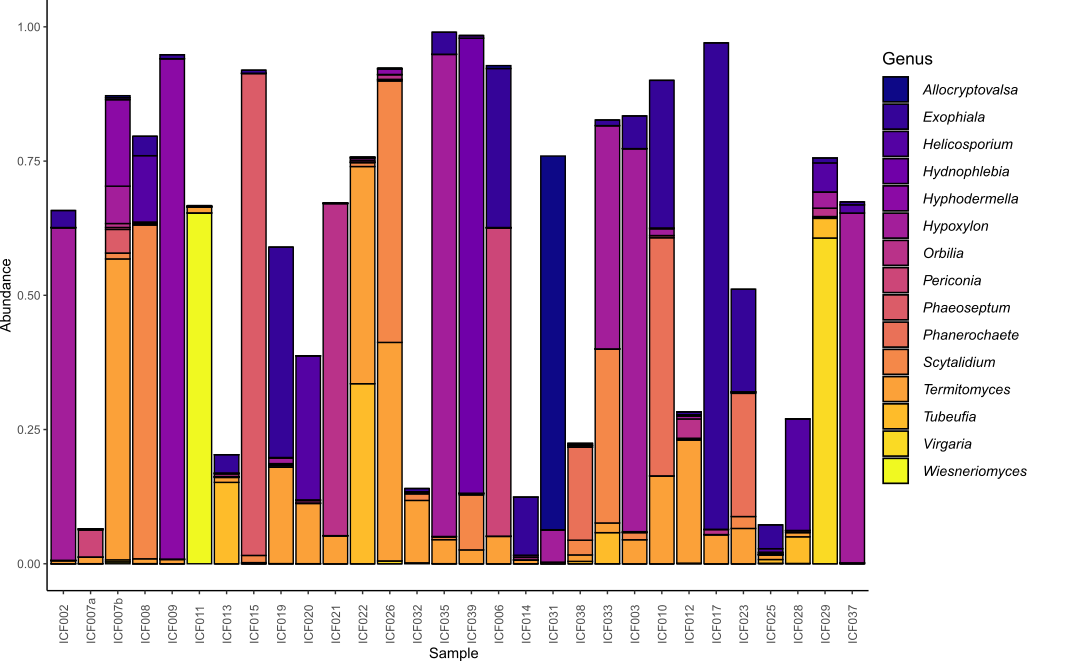


Figure S2: Relative abundance of the top 15 genera found in all collected foraging sites, including samples where no *Macrotermes bellicosus* foragers were found (see table S1 for details per foraging site, and table S2 for the full dataset).

**
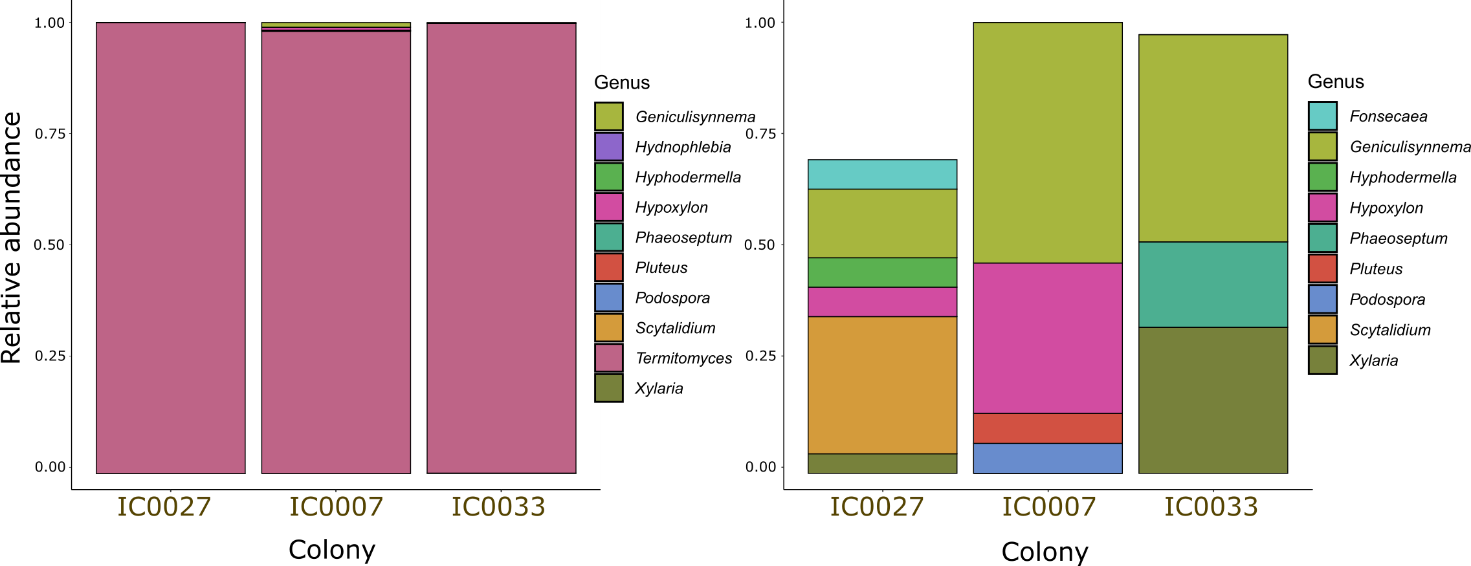
**

Figure S3: a) Relative abundance of the 10 most abundant genera present in fungus combs freshly collected from three colonies of *M. bellicosus*, showing vast dominance by *Termitomyces*. b) Relative abundance of the 10 most abundant non-*Termitomyces* genera present in freshly-collected combs of three colonies of *M. bellicosus* (see table S2 for the full dataset).
